# Independent effects of early life adversity on social cognitive function in patients with schizophrenia

**DOI:** 10.3389/fpsyt.2024.1343188

**Published:** 2024-03-05

**Authors:** Xing Peng, Wen-Peng Hou, Yu-Shen Ding, Qi Wang, Feng Li, Sha Sha, Chen-Chao Yu, Xiu-Jun Zhang, Fu-Chun Zhou, Chuan-Yue Wang

**Affiliations:** ^1^ School of Public Health, North China University of Science and Technology, Tangshan, China; ^2^ Beijing Key Laboratory of Mental Disorders, National Clinical Research Center for Mental Disorders & National Center for Mental Disorders, Beijing Anding Hospital, Capital Medical University, Beijing, China; ^3^ Advanced Innovation Center for Human Brain Protection, Capital Medical University, Beijing, China; ^4^ Department of Psychiatry, Fengtai Mental Health Center, Beijing, China; ^5^ Independent Researcher, Beijing, China

**Keywords:** early life adversity, childhood trauma, bullying, schizophrenia, social cognition

## Abstract

**Objective:**

The aim of this study was to investigate the impact of early life adversity on cognitive function in patients with schizophrenia, with a focus on social cognition (SC).

**Methods:**

Two groups of patients with schizophrenia were recruited and matched on sociodemographic and clinical characteristics. One group consisted of 32 patients with a history of childhood trauma (SCZ-ct), and the other group consisted of 30 patients without a history of childhood trauma (SCZ-nct). In addition, 39 healthy controls without a history of childhood trauma (HC-nct) were also recruited. The intelligence of the three groups was assessed using the Wechsler Abbreviated Scale of Intelligence (WAIS—RC) short version. The cognitive function evaluation was conducted using the MATRICS Consensus Cognitive Battery (MCCB), and early life adversity was measured using the Childhood Trauma Questionnaire-Short Form (CTQ) and Bullying Scale for Adults (BSA).

**Results:**

Patients with schizophrenia endosed significantly higher scores on the CTQ (F=67.61, p<0.001) and BSA (F=9.84, p<0.001) compared to the HC-nct. Analysis of covariance (ANCOVA) and *post-hoc* analyses revealed that SCZ-ct (F=11.20, p<0.001) exhibited the most pronounced cognitive impairment among the three groups, as indicated in MCCB total scores and in the domain score of SC. CTQ exhibited a negative correlation with MCCB (r=-0.405, p< 0.001); SC was negatively correlated with physical abuse (PA) of CTQ (r=-0.271, p=0.030) and emotional abuse (EA) of BSA (r=-0.265, p=0.034) in the whole patient sample. Higher SC performance was significantly predicted by CT_total (Beta =-0.582, p<0.001, 95% CI -0.96-0.46), and years of education (Beta=0.260, p =0.014, 95% CI 0.20-1.75) in schizophrenia.

**Conclusions:**

Besides familial trauma, schizophrenia patients appear to have a higher likelihood of experiencing bullying in their early life. These experiences seem to contribute significantly to their severe impairments in SC.

## 1 Introduction

Schizophrenia is recognized as a severe and complex mental disorder (1, 2) that is often associated with impairments in social cognitive (SC) function (3–5). Patients with schizophrenia may experience difficulties in accurately understanding the emotions, intentions, and social norms of others (6, 7). These issues frequently lead to conflicts and instances of aggressive behavior between patients with schizophrenia and their close caregivers, resulting in strained interpersonal relationships (8, 9). Consequently, these factors disrupt normal social interactions and significantly impact the individual’s quality of life and career development (10, 11).

The pathogenic mechanisms underlying schizophrenia are not yet fully elucidated. Besides genetic dispositions, previous research has focused on sociopsychological factors (5, 12). Among these factors, childhood trauma (CT) has been proposed as a potential risk factor for schizophrenia onset (13–15). This includes experiences such as physical abuse, emotional abuse, sexual abuse, physical neglect, and emotional neglect (14, 16, 17). These traumatic experiences may not only potentially heighten the risk of adolescents exhibiting psychotic symptoms (17–19) but also increase their vulnerability to the development of schizophrenia (20).

Furthermore, experiences of bullying during adolescence may serve as another potential factor in the onset of schizophrenia (12, 21). A study found that boys who frequently engage in bullying behaviors, or those who frequently find themselves the victims of such behaviors, are more susceptible to developing schizophrenia in early adulthood (22). This finding is further substantiated by a longitudinal investigation involving 4,720 participants, which concluded that regardless of whether one is a perpetrator or victim of bullying, experiencing bullying during adolescence increases the risk of developing mental disorders (23). This body of evidence points toward a discernable connection between bullying and mental illness, specifically schizophrenia.

The cognitive function of patients with schizophrenia is impaired, as established by previous research (3, 4, 24, 25). However, limited studies have implemented the threshold value of the Childhood Trauma Questionnaire-Short Form (CTQ) to differentiate patients with schizophrenia who have experienced childhood trauma from those who have not and to investigate potential differences in cognitive functioning between these two groups. This is particularly significant in the context of their social cognition function, which significantly impacts their quality of life and successful reintegration into society. Existing literature suggests that exposure to social adversity during childhood may contribute to the formation of negative internal working models, selective attention toward emotional stimuli and greater difficulties with emotional self-regulation (26). Moreover, studies have revealed that CT can affect an individual’s social cognitive functioning, particularly in terms of their intimate relationship with their mother (5). The presence of physical neglect significantly influences the intimacy between patients with schizophrenia and their parents, standing as the most potent predictor for their challenges in emotional recognition (5).

The purpose of the present study was to explore the effects of self-reported CT and bullying on the cognitive function of schizophrenia patients, with a focus on social cognition. The hypotheses formulated for this study were as follows (1): Patients with schizophrenia are likely to have endured more adversities during their early life - as quantified by the CTQ and the Bullying Scale for Adults (BSA) - in comparison to those without any such diagnosis (2); Experiencing adversities during early life could potentially impair cognitive function, with a particular impact on SC.

## 2 Methods

### 2.1 Participant enrollment

A total of 32 schizophrenia patients with a history of childhood trauma (SCZ-ct), 30 schizophrenia patients without a history of childhood trauma (SCZ-nct), and 39 healthy controls (HC-nct) without childhood trauma or any form of mental illness were recruited for this study (see **Table 1**). Eligible patients were referred to the study from both inpatient and outpatient departments at Beijing Anding Hospital, Capital Medical University. The inclusion criteria for the study were as follows (1): a diagnosis of schizophrenia according to the criteria of the Diagnostic and Statistical Manual of Mental Disorders, Fifth Edition (DSM-5) (2); a minimum of 9 years of education (3), an IQ of at least 80 (4), native Chinese speakers, and (5) aged between 18 and 60. Patients were excluded from the study if they had a history of substance abuse or had undergone any form of brain stimulation therapies within the 3 months prior to the study. The HCs were recruited from nearby communities. Prior to their participation, informed consent was obtained from all participants. The study protocol was approved by the Ethics Committee of Beijing Anding Hospital, Capital Medical University, and North China University of Science and Technology.

**Table 1:** Sociodemographic characteristics and early life adversities among the three groups of participants ( $\bar{x}\pm S$ ).

Variables	SCZ-ct (n=32)	SCZ-nct (n=30)	HC-nct (n=39)	*t/F/χ* ^2^	*P*
**Gender (male/female)**	20/18	23/12	18/21	2.92	0.230
**Age (years)**	30.43 ± 8.70	30.94 ± 9.24	28.97 ± 6.92	1.73	0.841
**Years of education**	14.61 ± 3.72	14.40 ± 3.67	16.00 ± 2.38	2.63	0.080
**IQ**	99.91 ± 13.47	108.81 ± 12.19	118.46 ± 9.92	22.83	<0.001
**Duration of illness (months)**	93.88 ± 87.76	85.14 ± 75.83	–	0.44	0.659
**OLZeq (mg)**	12.87 ± 7.15	13.34 ± 8.59	–	-0.22	0.830
**PANSS**	63.46 ± 19.27	64.14 ± 13.43	–	-0.17	0.863
**Positive**	16.62 ± 6.48	17.54 ± 5.01	–	-0.65	0.519
**Negative**	17.14 ± 7.28	16.60 ± 6.10	–	0.34	0.737
**General Psychopathology**	29.70 ± 8.76	30.00 ± 6.39	–	-0.16	0.870
**CT_total**	46.92 ± 9.85	31.54 ± 4.83	29.64 ± 5.39	67.61	<0.001
**CT_EA**	8.13 ± 3.03	6.23 ± 1.68	6.36 ± 1.93	6.54	0.002
**CT_PA**	7.11 ± 2.92	5.45 ± 0.85	5.41 ± 0.75	10.59	<0.001
**CT_SA**	6.42 ± 2.32	5.37 ± 0.97	5.17 ± 0.45	18.77	0.001
**CT_EN**	14.92 ± 5.43	8.00 ± 2.97	7.05 ± 2.33	8.41	<0.001
**CT_PN**	10.34 ± 3.20	6.08 ± 1.36	5.64 ± 1.16	7.80	0.001
**BSA**	13.66 ± 10.21	11.00 ± 10.87	4.59 ± 6.05	9.84	<0.001
**EA**	7.74 ± 5.65	5.89 ± 6.07	2.67 ± 3.09	9.88	<0.001
**ID**	3.71 ± 4.15	3.60 ± 4.94	1.10 ± 2.11	5.54	0.005
**PA**	2.05 ± 2.36	1.26 ± 1.52	0.59 ± 1.50	6.08	0.003
**SH**	0.16 ± 0.49	0.26 ± 0.70	0.23 ± 0.87	0.19	0.832

SCZ-ct, schizophrenia patients with a history of childhood trauma; SCZ-nct, schizophrenia patients without a history of childhood trauma; HC-nct, healthy controls without a history of childhood trauma; IQ, Intelligence Quotient; OLZeq, Olanzapine equivalent dose of antipsychotics; PANSS, Positive and Negative Syndrome Scale; CT_total, total childhood trauma; CT_EA, Emotional Abuse; CT_PA, Physical Abuse; CT_SA, Sexual Abuse; CT_EN, Emotional Neglect; CT_PN, Physical Neglect; BSA, Bullying Scale for Adults; EA, Emotional Abuse; ID, Interpersonal Difficulties; PA, Physical Abuse; SH, Sexual Harassment.The symbol "–" means inapplicable.

### 2.2 Assessment

#### 2.2.1 Clinical Assessments

The diagnosis of schizophrenia in patients was conducted using the Chinese version of the MINI International Neuropsychiatric Interview (MINI) (7.0.2) (27–29). The Chinese version of the Positive and Negative Syndrome Scale (PANSS) (30, 31) was administered to assess the clinical symptoms of patients with schizophrenia.

#### 2.2.2 Cognitive Assessments

The Wechsler Abbreviated Scale of Intelligence (WAIS—RC) short version (32, 33) was utilized to evaluate the intelligence of the three groups.

The MATRICS Consensus Cognitive Battery (MCCB) (34–37) was administered to measure cognitive function across the three groups. The MCCB has been developed for assessing the cognitive functions of schizophrenia and related disorders and has exhibited good reliability and small practice effects in a wide range of clinical studies in individuals with psychosis. There are 10 subtests in the Chinese version of MCCB (35) which are organized into the following 7 domains: 1. Speed of Processing (SP): Trail Making Test: Part A (TMT), Brief Assessment of Cognition in Schizophrenia (BACS): Symbol Coding, and Category Fluency Test: Animal naming (Fluency); 2. Attention/Vigilance (A/V): Continuous Performance Test-Identical Pairs (CPT-IP); 3. Working Memory (WM): Wechsler Memory Scale—Third Edition (WMS-III): Spatial Span; 4. Verbal Learning (VEL): Hopkins Verbal Learning Test—Revised (HVLT-R); 5. Visual Learning (VIL): Brief Visuospatial Memory Test-Revised (BVMT-R); 6. Reasoning and Problem Solving (RPS): Neuropsychological Assessment Battery (NAB): Mazes; 7. Social Cognition (SC): Mayer–Salovey–Caruso Emotional Intelligence Test (MSCEIT): Managing Emotions.

In particular, the Mayer–Salovey–Caruso Emotional Intelligence Test (MSCEIT) serves as a social cognitive measurement that evaluates the participant’s capacity to perceive, use, understand, and regulate emotions through a series of questions. The MSCEIT applies questions based on everyday situations to gauge the ability with which individuals navigate social tasks, interpret facial expressions, and address emotionally-charged problems (38, 39).

### 2.3 The childhood trauma questionnaire-short form

The CTQ developed by Bernstein and Fink (40, 41) was utilized in our study. The CTQ is a widely recognized self-assessment tool specifically designed to retrospectively evaluate experiences of maltreatment and neglect during childhood. This tool is composed of 28 items, each rated on a five-point Likert scale, with response options extending from “0” (indicating ‘never’) to “4” (indicating ‘very often’).

The CTQ assigns different threshold scores for each of its subscales: emotional abuse (EA) has a threshold of 13, physical abuse (PA) is set at 10, sexual abuse (SA) requires a minimum of 8, emotional neglect (EN) is set at 15, and physical neglect (PN) has a threshold of 10 (42). Patients with scores meeting or exceeding the designated threshold on any dimension are classified as SCZ-ct (42).

### 2.4 The Bullying Scale for Adults

The BSA, derived from the Bully Survey (43), was developed as a modified version to assess adults’ past experiences of bullying (44). The BSA comprises three distinct parts. Part A concentrates on personal experiences related to bullying. It includes 13 items representing four types of bullying behavior, namely Emotional Abuse (EA), Interpersonal Difficulties (ID), Physical Abuse (PA), and Sexual Harassment (SH). Participants rate each item using a five-point Likert scale, ranging from “0” (never) to “4” (always), with an additional option of “don’t know”. If a score other than “0” is selected, respondents are required to provide more detailed information regarding the perpetrator, time, and duration of the incidents. Part B examines the personal consequences of bullying, encompassing six items measured on a five-point Likert scale (“0-Never a problem” to “4-Always a problem”). Last, Part C includes two items that inquire about the experience of acting as a bullying perpetrator. Our previous study yielded favorable outcomes in terms of reliability and validity in the Chinese version of the BSA (45).

### 2.5 Statistical analysis

Statistical analysis was performed using SPSS 22.0 software. Categorical data were analyzed by the chi-square test. Analysis of covariance (ANCOVA) was employed to compare the differences in cognitive function among the groups, with IQ as a covariate. To understand the relationships between the variables, the Pearson correlation was applied. To investigate the influencing factors of SC, relevant sociodemographic and clinical variables were entered as independent variables in a stepwise multiple linear regression analysis. Numerical data are expressed as the mean ± standard deviation d. Multiple comparisons were adjusted using the Bonferroni correction, P<0.05 means the difference is statistically significant, and a two-tailed test is adopted.

## 3 Results

### 3.1 Sociodemographic characteristics and early life adversities

Six patients were excluded from the SCZ-ct group and 5 patients were excluded from the SCZ-nct group due to low IQ scores (IQ<80). Consequently, 32 SCZ-ct patients, 30 SCZ-nct patients and 39 heathy controls were included in the analyses. There was a significant difference in the scores of IQ among the three groups (**Table 1**). *Post-hoc* analyses revealed that individuals in SCZ-ct group had the lowest IQ scores (**Supplementary Table 1**). Apart from IQ, no statistically significant differences were observed in sociodemographic among the three groups or in the clinical features between the two groups of patients.

The BSA total score and dimensional scores were compared between SCZ-ct, SCZ-nct, and HC. One-way ANOVA and *post hoc* analyses showed that SCZ-ct had significantly higher scores in BSA (F=9.84, p<0.001), EA (F=9.88, p<0.001), ID (F=5.54, p =0.005), and PA (F=6.08, p =0.003) than HC-nct. SCZ-ct and SCZ-nct had comparable scores in BSA and all dimensional scores.

In the whole patient sample, the mean scores of BSA (F=9.84, p< 0.001) and ID (F=5.54, p=0.005) were significantly higher than that of HC-nct (**Table 1**; **Supplementary Figure 1**).

### 3.2 Cognitive result

**Figure 1:**
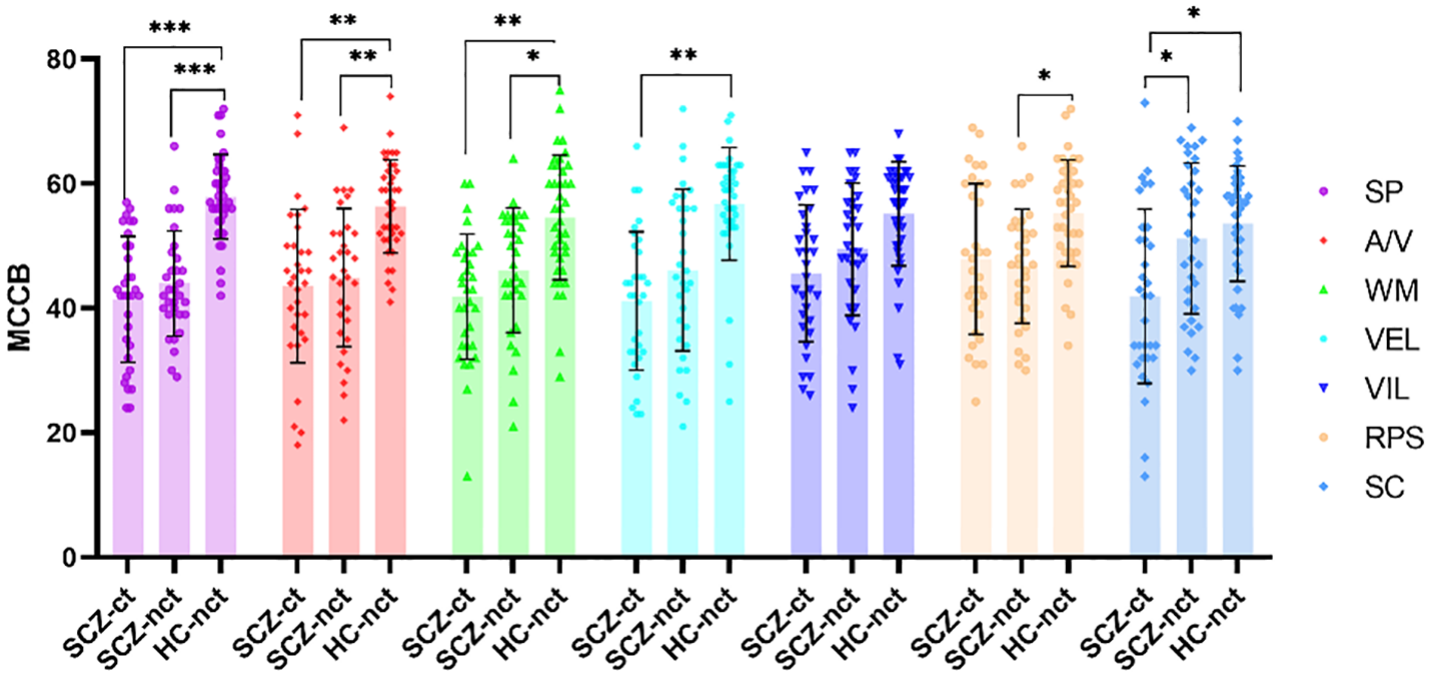
Comparisons of MCCB among three groups of participants. SCZ-ct, schizophrenia patients with a history of childhood trauma; SCZ-nct, schizophrenia patients without a history of childhood trauma; HC-nct, healthy controls without a history of childhood trauma; MCCB, MATRICS Consensus Cognitive Battery; SP, Speed of Processing; A/V, Attention/Vigilance; WM: Working Memory; VEL, Verbal Learning; VIL, Visual Learning; RPS, Reasoning and Problem Solving; SC, Social Cognition. *p<0.05; **p<0.01; ***p<0.001.

Analysis of Covariance (ANCOVA) was conducted with IQ as a covariate, revealing significant differences in MCCB total score among the three groups (F=11.20, p<0.001). *Post-hoc* analyses indicated that SCZ-ct exhibited the most severe cognitive impairment with the lowest scores. Similar results were observed in several domain scores, including SP (F=17.62, p<0.001), A/V (F=7.28, p<0.001), WM (F=5.02, p=0.008), VEL (F=5.68, p=0.005), RPS (F=3.63, p=0.030), and SC (F=4.17, p=0.018). In terms of SC, the score of SCZ-nct was not significantly different from that of HC-nct (p=1.000) but was significantly higher than that of SCZ-ct (p=0.030) (**Table 2**; **Figure 1**).

**Table 2:** Comparisons of MCCB scores among three groups of participants ( $\bar{\text{x}}$ ±*S*).

Variables	SCZ-ct (n=32)	SCZ-nct (n=30)	HC-nct (n=39)	*F*	*P*	*η^2^*	*Post-hoc*		
							*p*, Bonferroni-adjusted		
							SCZ-ct vs. SCZ-nct	SCZ-ct vs. HC-nct	SCZ-nct vs. HC-nct
**MCCB**	43.70 ± 1.77	46.76 ± 1.67	55.16 ± 1.60	11.20	<0.001	0.188	0.756	<0.001	0.001
**SP**	44.59 ± 1.45	44.65 ± 1.37	55.01 ± 1.31	17.62	<0.001	0.266	1.000	<0.001	<0.001
**A/V**	45.44 ± 1.95	45.86 ± 1.84	54.64 ± 1.77	7.28	<0.001	0.130	1.000	0.005	0.003
**WM**	44.13 ± 1.86	45.91 ± 1.76	52.29 ± 1.69	5.02	0.008	0.094	1.000	0.013	0.033
**VEL**	44.47 ± 1.95	47.60 ± 1.85	53.80 ± 1.77	5.68	0.005	0.105	0.725	0.004	0.056
**VIL**	48.30 ± 1.81	50.36 ± 1.71	52.70 ± 1.64	1.40	0.253	0.028	1.000	0.296	0.996
**RPS**	49.42 ± 1.90	47.19 ± 1.79	53.90 ± 1.72	3.63	0.030	0.070	1.000	0.322	0.026
**SC**	43.85 ± 2.23	51.83 ± 2.11	51.87 ± 2.03	4.17	0.018	0.079	0.030	0.046	1.000

MCCB, SCZ-ct, schizophrenia patients with a history of childhood trauma; SCZ-nct, schizophrenia patients without a history of childhood trauma; HC-nct, healthy controls without a history of childhood trauma; MATRICS Consensus Cognitive Battery; SP, Speed of Processing; A/V, Attention/Vigilance; WM, Working Memory; VEL, Verbal Learning; VIL, Visual Learning; RPS, Reasoning and Problem Solving; SC, Social Cognition.

### 3.3 Correlation between CT, BSA and MCCB results

In the whole sample of schizophrenia patients, CTQ showed a significant negative correlation with MCCB (r = -0.405, p< 0.001), and CTQ_PA exhibited a negative association with the SC scores (r = -0.271, p = 0.030) on the MCCB. Furthermore, the BSA_EA exhibited a negative correlation with the SC scores (r = -0.265, p = 0.034) on the MCCB (**Supplementary Table 2**).

### 3.4 Exploring factors that predict SC function

In the stepwise linear regression analyses, SC was entered as the dependent variable, and the scores of CTQ, CTQ_PA, BSA_EA, and education were entered as independent variables. The results suggested that higher SC score was significantly predicted by CTQ (Beta =-0.582, p<0.001, 95% CI -0.96-0.46) and education (Beta = 0.260, p =0.014, 95% CI 0.20-1.75) in schizophrenia patients (**Table 3**).

**Table 3:** Results of the stepwise multiple regression analysis (patients; n=62).

Social cognition	Predictor	Beta	p-value	95% CI
**Adjusted R^2 ^= 0.360;** **F**_(2, 59)_ **= 18.19; p<0.001**	**CT_total**	-0.582	<0.001	-0.96, -0.46
	**Years of education**	0.260	0.014	0.20,1.75

CT_total, total childhood trauma; CT_PA, Physical Abuse; BSA_EA, Emotional Abuse.

## 4 Discussion

To the best of our knowledge, this is the first study in which the CTQ, BSA, and MCCB have been employed to examine the impact of early life adversity on cognitive functioning, particularly in the domain of SC, in patients with schizophrenia. The findings of the study have confirmed our hypothesis that, compared to healthy controls, patients with schizophrenia experience more early life adversity, including bullying. These early life adversities are associated with poorer cognitive functioning, especially SC, which has been considered to be closely related to quality of life and occupational development of the patients.

As hypothesized, patients with schizophrenia had significantly higher scores on the CTQ and BSA than healthy controls, which means that patients with schizophrenia experience more early life adversities. The study findings are in line with previous research studies. A meta-analysis of 36 studies (46) revealed that, compared to healthy controls, the odds ratio (OR) for CT in patients with schizophrenia was 2.78 (95% CI: 2.34-3.31). Specific forms of trauma showed an OR of 2.90 (95% CI: 1.71-4.92) for neglect, 3.40 (95% CI: 2.06-5.62) for emotional abuse, 2.95 (95% CI: 2.25-3.88) for physical abuse, and 2.38 (95% CI: 1.98-2.87) for sexual abuse. Another study (47) indicated that patients with schizophrenia had higher scores on measures of sexual abuse than healthy controls. According to a prospective cohort study (23) of 4720 children aged 8 to 11 years, children who reported bullying had a higher likelihood of developing a mental illness by age 18 than children who did not (OR=2.4, 95% CI: 1.6-3.4). A case‒control study (12) also found that schizophrenia patients were bullied twice as much as healthy controls. According to revictimization theory (48–50), individuals who experience childhood trauma may be more likely to become targets of bullying and attacks and are more likely to experience further trauma. A longitudinal study (51) validated this theory, as 213 (11.1%) out of 1915 young adults who experienced sexual abuse before adulthood reported experiencing sexual abuse again in adulthood.

SCZ-ct group was observed to have the lowest IQ scores among the three groups in the current study. This finding aligns with a 6-year follow-up study involving 1,119 patients with schizophrenia spectrum disorders. The study revealed that patients with schizophrenia spectrum disorders who had a history of childhood trauma exhibited the least progress in intellectual abilities, suggesting the lowest learning effect (52). Similar results were found in a study examining the impact of childhood trauma on intellectual functioning in patients with bipolar disorder, indicating that the presence of childhood trauma further impairs their intellectual abilities (53). This may be attributed to the negative effect of trauma on neural development, leading to alterations in brain structure and function (7, 54, 55).

In terms of SC, patients with schizophrenia have difficulty accurately understanding others’ emotions, intentions, and social signals when communicating with caregivers (5) or during social interactions. They may misunderstand others’ facial expressions, language, and nonverbal cues (6, 56), leading to poor communication and interpersonal tension. The results confirm our hypothesis that SC was significantly associated with childhood trauma, as indicated in the stepwise linear regression analyses. We found that the factors of “physical abuse” in CTQ and “emotional abuse” in BSA (bullying scales for adult) were significantly anti-correlated with social cognitive function (r = -0.271 and -0.265, respectively). In the multivariate regression, no specific type of trauma or bullying experience were identified to be independent predictors for social cognition, and the only contributor was the total score of CTQ (Beta=-0.582). This suggests that the social cognition impairments would better be explained by a cumulative effects of various kinds of childhood adversities.

Previous studies have shown that cognitive function is impaired in patients with schizophrenia (3, 4, 24, 25), with CT playing an important role in SC impairment in schizophrenia (5, 7, 26, 56–58). One study showed that schizophrenia patients had significantly lower scores in parental relationships and SC than healthy controls, with physical neglect being the strongest predictor of emotional recognition impairment. Good parental relationships were found to alleviate this emotional problem (5). Another survey involving 204 children aged 8-11 found that victims of bullying had difficulty in some moral judgment tasks (59), indicating that bullying has a negative impact on individuals’ SC. Furthermore, CT was found not only to affect the SC of schizophrenia patients but also to contribute to the cognitive impairment of bipolar disorder patients, specifically in working memory and executive function (60). A comprehensive review of 1,723 mood disorders and 797 healthy controls also confirmed that CT has a certain impact on the cognitive and executive functions of mood disorders (61).

Schizophrenia is a complex and severe mental disorder that involves various biological, psychological, social, and environmental factors (1, 2, 62–65), and CT increases the risk of developing this illness. Long-term and repeated exposure to negative environments, such as trauma and bullying, disrupts the balance of neurohormones (particularly glucocorticoids) (66), which can affect neuronal development and connectivity while altering epigenetic modifications (67). This process also impacts synaptic pruning during critical periods of early brain development (68, 69), as early life is a crucial period for individual brain development (53, 70, 71). Therefore, early-life adversities, including childhood trauma, disturb the balance of neurohormone secretion and lead to structural changes in the brain. These structural alterations can affect the cognitive functioning of patients with schizophrenia.

Notably, we found that the SC scores of SCZ-ct were significantly lower than those of SCZ-nct, while there was no significant difference in neural cognition dimensions (58, 72). It appears that CT independently contributes to the shaping of SC. Childhood is considered an important stage for both individual neural development and SC development (53, 70, 71, 73). During this period, the social environment in which children exist has a profound impact on their SC and behavioral patterns (74). According to Bowlby’s attachment theory (75), developing a secure attachment with a caregiver, especially one related to the mother, during childhood enables individuals to form a positive and secure “internal working model” in their interactions with others. This secure attachment relationship is an important prerequisite for successful social interactions. In contrast, experiencing adverse traumatic events during childhood, such as low levels of care and neglect, hinder the development of a secure attachment relationship, leading to difficulties in trusting others, exhibiting poor adaptability to the environment in adulthood (76), and even manifesting psychotic symptoms. Bowlby’s attachment theory (75) provides a cognitive-developmental framework for understanding the potential mechanisms by which early-life adversity may impact SC abilities later in life.

### 4.1 Limitations

There are some limitations to this study that need to be mentioned. First, the sample size employed was relatively small, thereby resulting in a decrease in statistical power. For example, no effort was made to match the intelligence levels among the three groups, although a covariance analysis was undertaken to mitigate the effects of intellectual impairment on the differences in cognitive function. Second, the study’s reliance on the self-assessment retrospective nature of the childhood trauma questionnaire introduces the possibility of potential recall biases in the patients’ reported experiences of childhood trauma. Furthermore, SC is a complex set of mental abilities that involve many aspects. A limitation of the MCCB is that there is only one test in the domain of SC. Future studies should consider the utilization of an integrated social cognition battery to assess various aspects of this cognitive function. Last but not least, additional variables such as genetic factors and psychiatric comorbidities could potentially confound the result but were not collected in the present study. These factors should be taken into consideration for a more comprehensive interpretation of the results in the future.

## 5 Conclusion

In addition to familial trauma, patients with schizophrenia are more susceptible to experiencing bullying during their early life. Both factors contribute significantly to the exacerbation of their social cognitive impairments. Our research enhances the understanding of the impact of early life adversity on cognitive functioning, specifically social cognitive function, in individuals with schizophrenia.

Moreover, the findings of this study offer valuable insights for the development of innovative psychological interventions. These interventions aim at alleviating the long-term repercussions of early life adversity for individuals with schizophrenia. This underscores not only the importance of childhood trauma experiences, which are currently considered during clinical assessments and treatment plans but also elevates the significance of bullying experiences in individuals with schizophrenia.

Therefore, to prevent and mitigate the lasting effects of childhood trauma and bullying on individual development and potential psychiatric disorders in individuals with schizophrenia, the development of new social-psychological intervention strategies is essential. These would facilitate their recovery and social reintegration.

## Data availability statement

The original contributions presented in the study are included in the article/**Supplementary Material**, further inquiries can be directed to the corresponding authors.

## Ethics statement

The studies involving humans were approved by the Medical Ethics Committee of North China University of Science and Technology and the Ethics Committee of Beijing Anding Hospital, Capital Medical University. The studies were conducted in accordance with the local legislation and institutional requirements. The participants provided their written informed consent to participate in this study.

## Author contributions

XP: Conceptualization, Data curation, Formal analysis, Investigation, Methodology, Visualization, Writing – original draft, Writing – review & editing. W-PH: Writing – review & editing, Writing – original draft. Y-SD: Writing – review & editing, Writing – original draft. QW: Writing – review & editing, Data curation. FL: Writing – review & editing, Funding acquisition. SS: Writing – review & editing. C-CY: Writing – review & editing. X-JZ: Conceptualization, Funding acquisition, Project administration, Resources, Supervision, Writing – review & editing. F-CZ: Conceptualization, Funding acquisition, Project administration, Resources, Supervision, Writing – review & editing. C-YW: Conceptualization, Project administration, Resources, Supervision, Writing – review & editing.
