# Supplementary material for: Independent effects of early life adversity on social cognitive function in patients with schizophrenia
Source: Front Psychiatry. 2024 Mar 5;15:1343188. doi: 10.3389/fpsyt.2024.1343188 (PMC10948615; doi:10.3389/fpsyt.2024.1343188)
Supplement: Supplementary file 1 [file DataSheet_1.docx]

Supplementary Material

**Supplementary Table**

**1.Intelligence Result**

The outcomes of the One-Way ANOVA analysis exposed statistically significant disparities in intelligence scores (F=22.83, p < 0.001) among the three groups. Similarly, these significant differences were also evident across each domain of intelligence. SCZ-ct exhibited the lowest intelligence scores (See Supplementary Table 1).

**Supplementary Table 1** Difference analysis of WAIS—RC among three groups of subjects ($\bar{x}\pm S$)

| **Variables** | **SCZ-ct**  **(n=38)** | **SCZ-nct**  **(n=35)** | **HC-nct**  **(n=39)** | ***F*** | ***P*** | **Post-hoc** | | |
| --- | --- | --- | --- | --- | --- | --- | --- | --- |
|  |  |  |  |  |  | ***P*, Bonferroni-adjusted** | | |
|  |  |  |  |  |  | **SCZ-ct**  **vs.**  **SCZ-nct** | **SCZ-ct**  **vs.**  **HC-nct** | **SCZ-nct**  **vs.**  **HC-nct** |
| **IQ** | 99.91±13.47 | 108.81±12.19 | 118.46±9.92 | 22.83 | ＜0.001 | 0.002 | ＜0.001 | 0.001 |
| **IF** | 9.78±2.93 | 12.03±2.54 | 12.80±2.67 | 12.23 | ＜0.001 | 0.001 | ＜0.001 | 0.238 |
| **SL** | 11.33±2.29 | 12.30±2.52 | 13.74±2.00 | 10.74 | ＜0.001 | 0.079 | ＜0.001 | 0.008 |
| **PC** | 9.42±6.47 | 9.12±3.60 | 11.26±2.84 | 2.39 | 0.097 | 0.789 | 0.084 | 0.050 |
| **BD** | 10.31±6.00 | 11.57±2.67 | 14.26±5.36 | 6.23 | 0.003 | 0.290 | 0.010 | 0.024 |

Abbreviations: SCZ-ct: schizophrenia patients with a history of childhood trauma; SCZ-nct: schizophrenia patients without a history of childhood trauma; HC-nct: healthy controls without a history of childhood trauma; WAIS—RC: Wechsler Adult Intelligence Scale - Revised Chinese edition; IQ: Intelligence Quotient; IF: Information; SL: Similarities; PC: Picture Completion; BD: Block Design;

**2.** **Correlation between CT, C-BSA and MCCB result**

**Supplementary Table 2** Analysis of the correlation between childhood trauma, bullying and cognition function among three groups of subjects (*r*)

| **Variables** | **MCCB** | **SP** | **A/V** | **WM** | **VEL** | **VIL** | **RPS** | **SC** |
| --- | --- | --- | --- | --- | --- | --- | --- | --- |
| **CT_total** | -0.405*** | -0.083 | -0.083 | -0.067 | -0.022 | -0.099 | 0.142 | -0.137 |
| **CT_EA** | -0.234 | -0.234 | -0.062 | -0.162 | -0.151 | -0.149 | -0.134 | -0.235 |
| **CT_PA** | -0.238 | -0.145 | -0.016 | -0.194 | -0.202 | -0.310* | 0.001 | -0.271* |
| **CT_SA** | 0.055 | -0.012 | -0.075 | -0.030 | 0.058 | 0.098 | -0.021 | -0.082 |
| **CT_EN** | -0.29 | -0.002 | -0.081 | -0.021 | 0.096 | -0.024 | 0.235 | -0.033 |
| **CT_PN** | 0.081 | -0.014 | -0.154 | -0.030 | -0.006 | -0.001 | 0.184 | -0.062 |
| **BSA** | 0.058 | -0.076 | -0.030 | -0.066 | -0.073 | -0.080 | -0.074 | -0.169 |
| **EA** | -0.097 | -0.110 | 0.100 | -0.003 | -0.042 | 0.123 | -0.170 | -0.265* |
| **ID** | -0.001 | -0.028 | -0.064 | -0.351* | 0.153 | 0.214 | -0.043 | -0.014 |
| **PA** | -0.068 | -0.108 | 0.190 | -0.103 | 0.055 | 0.050 | -0.29 | -0.017 |
| **SH** | -0.020 | -0.113 | -0.072 | -0.058 | 0.065 | -0.054 | -0.29 | -0.050 |

Abbreviations: *：p＜0.05，**：p＜0.01；***：p＜0.001; CT_total: the total childhood trauma; CT_EA: Emotional Abuse; CT_PA: Physical Abuse; CT_SA: Sexual Abuse; CT_EN: Emotional Neglect; CT_PN: Physical Neglect; BSA: Bullying Scale for Adults; EA: Emotional Abuse; ID: Interpersonal Difficulties; PA: Physical Abuse; SH: Sexual Harassment; MCCB: MATRICS Consensus Cognitive Battery; SP: Speed of Processing A/V: Attention/Vigilance; WM: Working Memory; VEL: Verbal Learning; VIL: Visual Learning； RPS: Reasoning and Problem Solving; SC: Social Cognition；

## Supplementary Figures

**
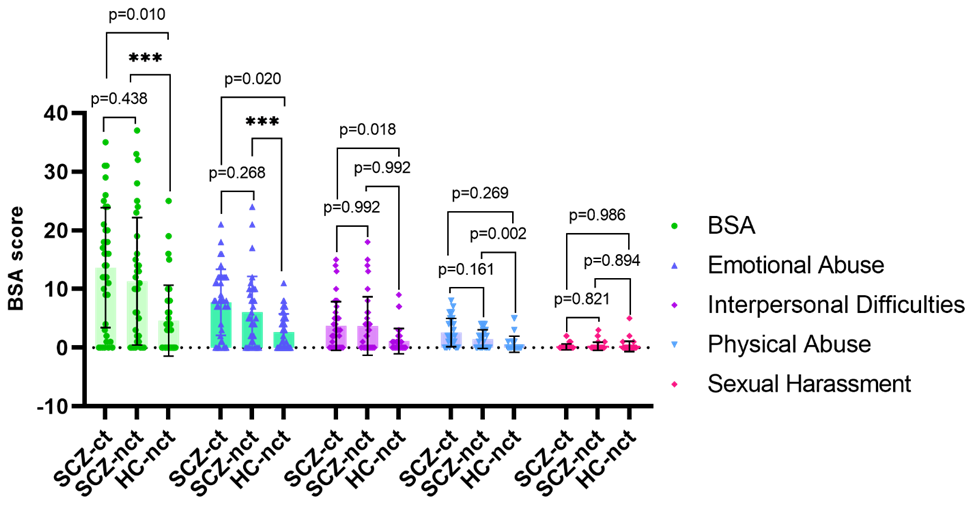
**

**Supplementary Figure 1.** Difference analysis of bullying among three groups of subjects

Abbreviations: BSA: Bullying Scale for Adults Chinese version; EA: Emotional Abuse; ID: Interpersonal Difficulties; PA: Physical Abuse; SH: Sexual Harassment;
